# Vascular and Neural Dysfunctions in Obese Zucker Rats: Effect of AVE7688

**DOI:** 10.1155/2009/912327

**Published:** 2009-06-07

**Authors:** Eric P. Davidson, Lawrence J. Coppey, Travis L. Kleinschmidt, Christine L. Oltman, Mark A. Yorek

**Affiliations:** ^1^Department of Veterans Affairs Iowa City Health Care System, University of Iowa, Iowa City, IA 52246, USA; ^2^Department of Internal Medicine, University of Iowa, Iowa City, IA 52246, USA

## Abstract

The purpose of this study was to determine whether AVE7688 a drug that inhibits both angiotensin converting enzyme and neutral endopeptidase activity protects vascular and nerve functions in an animal model of metabolic syndrome. Obese Zucker rats at 20 weeks of age were treated for 12 weeks with AVE7688. Vasodilation in epineurial arterioles was measured by videomicroscopy and nerve conduction velocity was measured following electrical stimulation. Treatment with AVE7688 improved vascular relaxation in response to acetylcholine and motor and sensory nerve conduction velocity. In obese Zucker rats superoxide levels and nitrotyrosine staining were elevated in the aorta and treatment corrected both conditions. Obese Zucker rats were hypoalgesic in response to a thermal stimulus and demonstrated signs of impaired tactile response and both conditions were significantly improved with treatment. Even though obese Zucker rats are normoglycemic vascular and neural dysfunctions develop with age and can be improved by treatment with AVE7688.

## 1. Introduction

Patients with impaired glucose tolerance, a feature of metabolic syndrome, have been described by some investigators as developing peripheral neuropathy with microvascular disease [1–4]. Also, patients with type 2 diabetes and metabolic syndrome have a higher incidence of diabetic neuropathy than diabetic patients without metabolic syndrome [4–7]. However, other investigators state that it is unclear whether impaired glucose tolerance is associated with diabetic sensorimotor polyneuropathy or chronic idiopathic axonal polyneuropathy and that some of the disparities may be due to differences in patient selection, assessment of glycemic exposure, and diabetic complications [8]. Nonetheless, there is a need for further study to determine whether patients with metabolic syndrome may be at increased risk for microvascular disease and peripheral neuropathy.

Previously, we demonstrated that obese Zucker rats, a model for metabolic syndrome, develop microvascular and neural deficits independently of hyperglycemia [9]. In obese Zucker rats, impaired relaxation in response to acetylcholine in epineurial arterioles and slowing of motor nerve conduction velocity were observed after 16–20 and 32 weeks of age, respectively, demonstrating that microvascular impairment preceded neural dysfunction. In the present study we sought to determine whether treatment of obese Zucker rats with AVE7688, a vasopeptidase inhibitor, for 12 weeks beginning at 20 weeks of age could improve microvascular dysfunction and prevent the slowing of nerve conduction velocity. Vasopeptidase inhibitors are a new class of drug that simultaneously inhibits neutral endopeptidase and angiotensin converting enzyme (ACE) activity [10]. Recent studies have shown increased expression of angiotensin II-forming enzymes in adipose tissue, and increased activity of the renin-angiotensin system has been implicated in the development of insulin resistance and type 2 diabetes [11]. Neutral endopeptidase is found in many tissues including vascular tissue and its activity is increased by fatty acids and glucose in human microvascular cells [12–16]. Neutral endopeptidase degrades many vasoactive peptides including natriuretic peptides, adrenomedullin, bradykinin, and calcitonin gene-related peptide [17, 18]. Therefore, inhibition of ACE and neutral endopeptidase activity would be expected to improve vascular function. In this regard, vascular conductance in the femoral artery of streptozotocin-induced diabetic rats to bradykinin was improved by a vasopeptidase inhibitor and we have shown that vasodilation by epineurial arterioles to acetylcholine and nerve function are improved in streptozotocin-induced diabetic rats and Zucker diabetic fatty rats (ZDF) treated with AVE7688 [19–21]. Vasopeptidase inhibitors have also been shown to be neuroprotective and prevent nephropathy in ZDF rats and decrease matrix metalloproteinases, AGE accumulation/formation in type 2 diabetes and improve wound healing [22–28]. Therefore, there is great potential for treatment of vascular and neural dysfunctions with vasopeptidase inhibitors; however, no information is available about the effect of these inhibitors in an animal model with features of metabolic syndrome.

## 2. Materials and Methods

Unless stated otherwise all chemicals used in these studies were obtained from Sigma Chemical Co. (St. Louis, MO). 

### 2.1. Animals

Male Zucker rats, obese and lean, were obtained at 6 weeks of age from Charles River Laboratories, Wilmington, MA. The lean animals were not genotyped and could have been either +/+ or +/− for the leptin receptor deletion. The animals were housed in a certified animal care facility and food (Harlan Teklad, no. 7001, Madison, WI) and water were provided ad libitum. All institutional and NIH guidelines for use of animals were followed.

At 20 weeks of age the obese Zucker rats were divided into two groups. One group was fed the standard chow diet. The second group was fed the standard chow diet containing 500 mg/kg AVE7688. Based on the amount of chow consumed the rats received approximately 30 mg/kg rat/day of AVE7688. The supplemented diet was prepared by thoroughly mixing the AVE7688 into the meal form of the diet for 1 hour. Afterwards, the diet was pelleted and dried in a vacuum oven set at 40°C overnight. The control diet was also prepared from meal. The treatment period lasted for 12 weeks.

### 2.2. Thermal Nociceptive and Tactile Response Threshold

The day before the terminal studies, tactile response threshold was measured with von Frey filaments using the modified up-down method [29–31]. Six hours later thermal nociceptive response in the hindpaw was measured using the Hargreaves method with instrumentation provided by IITC Life Science; Woodland Hills, CA (model 390G). These tests were performed when possible in a blind manner with the operator not knowing whether the obese Zucker rat was in the treatment or nontreated group. For the tactile response threshold measurement the rat was placed on a wire mesh grid and allowed to acclimate for 15 minutes. Afterwards, a von Frey filament was applied to the left hindpaw. Continuous pressure with the filament was applied for 5 seconds. If the animal lifted its paw, a positive response, then the next filament with less force was applied to the paw. If the animal did not respond, a negative response, then the next filament with increasing force was used. This process was continued until four measurements following the initial positive response were recorded. The results were quantified according to Chaplan et al. [29]. For the thermal nociceptive response measurement the rat was placed in the observation chamber on top of the thermal testing apparatus and allowed to acclimate to the warmed glass surface (30°C) and surroundings for a period of 15 minutes. The mobile heat source was maneuvered so that it was under the heal of the hindpaw and then activated, a process that activates a timer and locally warms the glass surface, when the rat withdrew its paw, the timer, and the heat source was turned off [32]. Following an initial recording, which was discarded, four measurements were made for each hindpaw, with a rest period of 5 minutes between each set of measurements. The mean of the measurements, reported in seconds, were used as a measure of the thermal nociceptive response.

On the day of the terminal experiments, rats were anesthetized with Nembutal i.p. (50 mg/kg, i.p., Abbott Laboratories, North Chicago, IL) and nonfasting blood glucose levels were determined with the use of glucose oxidase reagent strips (Lifescan Inc., Milpitas, CA). Serum samples were collected for determination of insulin levels using a Luminex 100 system. The insulin analyte kit was purchased from Upstate, Charlottesville, VA. Levels of serum-free fatty acid, triglyceride, free cholesterol, and ACE activity were determined using commercial kits from Roche Diagnostics, Mannheim, Germany; Sigma Chemical Co., St. Louis, MO; Bio Vision, Mountain View, CA; and ALPCO, diagnostics, Salem, NH; respectively. In all analyses the manufactures instructions were followed. Afterwards, motor and sensory nerve conduction velocity was determined and tissue containing the epineurial arterioles was collected.

### 2.3. Motor and Sensory Nerve Conduction Velocity

Motor nerve conduction velocity (MNCV) was determined as previously described using a noninvasive procedure in the sciatic-posterior tibial conducting system [33–36]. Sensory nerve conduction velocity (SNCV) was determined using the digital nerve to the second toe as described by Obrosova et al. [37]. The MNCV and SNCV were reported in meters per second.

### 2.4. Vascular Reactivity

Videomicroscopy was used to investigate in vitro vasodilatory responsiveness of arterioles vascularizing the region of the sciatic nerve as previously described [33–36]. The average lumen diameter of the epineurial arterioles used in these studies was about 100 *μ*m. Based on size and function these vessels can be considered to be microvessels. Cumulative concentration-response relationships were evaluated for acetylcholine (10^−8^–10^−4^ M) using vessels from each group of rats. At the end of each dose response determination a maximal dose of sodium nitroprusside (10^−4^ M) was added. Afterwards, papaverine (10^−5^ M) was added to determine maximal vasodilation.

### 2.5. Detection of Superoxide and Peroxynitrite

Using the aorta superoxide levels were measured by lucigenin-enhanced chemiluminescence as described previously [38]. Relative light units (RLU) were measured using a luminometer. Background activity was determined and subtracted and RLU was normalized to surface area.

One mechanism by which acetylcholine can mediate vascular relaxation in arterioles that provide circulation to the sciatic nerve is through the production of nitric oxide [39]. The chemistry of nitric oxide is complex, and several biochemical pathways other than nitric oxide production can influence nitric oxide bioactivity. For example, superoxide anion can interact with nitric oxide to form peroxynitrite [40]. This reaction reduces the efficacy of nitric oxide to act as a signal transduction agent. Peroxynitrite is a highly reactive intermediate known to nitrate protein tyrosine residues and cause cellular oxidative damage [40, 41]. To determine whether formation of superoxide by the aorta promotes the formation of peroxynitrite we measured 3-nitrotyrosine (3-nitrotyrosine is a stable biomarker of tissue peroxynitrite formation) in vessel sections from 32-week old lean and obese Zucker rats treated with or without AVE7688. Briefly, frozen tissue segments of aorta were cut into 10 *μ*m sections and then incubated in phosphate buffered saline solution containing 1% Triton X-100 and 0.1% bovine serum albumin for 30 minutes at room temperature. Afterwards, the samples were incubated overnight at 4°C in this buffer solution containing mouse antinitrotyrosine antibody (Upstate, Lake Placid, NY). After washing, the sections were incubated for 2 hours with Alexa Fluor 555 goat antimouse IgG (Molecular Probes, Eugene, OR). Sections were then rinsed and mounted with VectorShield. The labeled vessels derived from these studies were visualized with an Olympus IX71 inverted research microscope interfaced with a PC containing SimplePCI imaging software. Pixel intensity for nitrotyrosine immunostaining was determined for each vessel segment and averaged for each condition.

### 2.6. Data Analysis

The results are presented as mean ± SE. Comparisons between the groups for body weight, blood glucose, MNCV, SNCV, thermal nociception, tactile allodynia, serum TBARS, serum free fatty acid, triglyceride, cholesterol and insulin levels, serum ACE activity, aorta superoxide and nitrotyrosine levels were conducted using a one-way ANOVA and Newman-Keuls test for multiple comparisons and Bonferroni-Dunn test (Prism software; GraphPad, San Diego, CA). Concentration response curves for acetylcholine- and CGRP-induced relaxation were compared using a two-way repeated measures analysis of variance with autoregressive covariance structure using a proc mixed program of SAS [33–36]. Whenever significant interactions were noted specific treatment-dose-effects were analyzed using a Bonferroni-Dunn test. A *P* value of less 0.05 was considered significant.

## 3. Results

Data in Table 1 demonstrate that lean and obese Zucker rats weighed about the same at 6 weeks of age. However, by 32 weeks of age obese Zucker rats weighed significantly more then the lean control rats and treatment with AVE7688 did not influence weight gain in obese Zucker rats. Blood glucose levels were not significantly different between lean rats and treated or untreated obese Zucker rats. Insulin levels were significantly increased in obese Zucker rats compared to lean rats. Furthermore, treating obese Zucker rats with AVE7688 increased insulin levels. Free cholesterol levels tended to be higher in both untreated and treated obese Zucker rats but compared to the lean controls this difference did not reach statistical significance. Serum triglyceride and free fatty acid levels were significantly increased in obese Zucker rats compared to lean rats and lowered with treatment of obese Zucker rats with AVE7688. Serum ACE activity was also significantly increased in obese Zucker rats and lowered with treatment of AVE7688.

**Figure 1:**
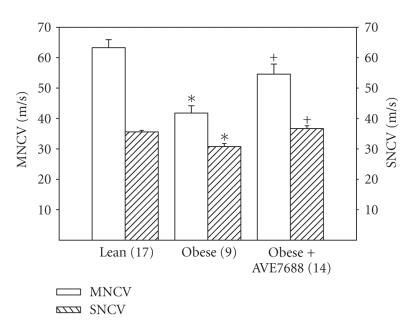
Determination of the effect of treatment of obese Zucker rats with AVE7688 for 12 weeks at 20 weeks of age on motor and sensory nerve conduction velocity. Data are presented as the mean ± SEM in meters/second. The number of experimental determinations is presented in parenthesis. *P < .05, compared to lean rats, ^+^ *P* < .05 compared to untreated obese Zucker rats.

Data in Figure 1 demonstrate that in obese Zucker rats at 32 weeks of age motor and sensory nerve conduction velocity was significantly decreased compared to lean control rats. Treating obese Zucker rats for 12 weeks with AVE7688 prevented the slowing of nerve conduction velocity.

Data in Table 1 demonstrate that after 32 weeks of age obese Zucker rats develop thermal hypoalgesia and have an impaired tactile response threshold. Treating obese Zucker rats with AVE7688 significantly improved the impairment in pain perception.

**Figure 2:**
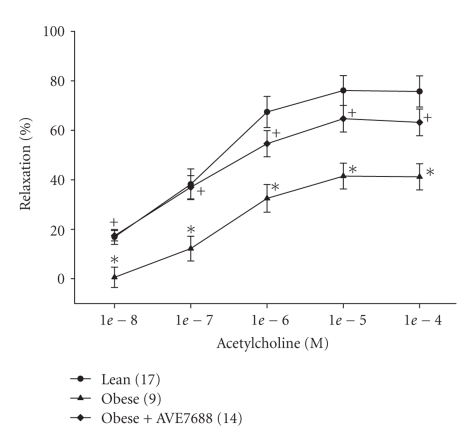
Determination of the effect of treatment of obese Zucker rats with AVE7688 for 12 weeks at 20 weeks of age on acetylcholine-mediated vascular relaxation of epineurial arterioles of the sciatic nerve. Pressurized arterioles (40 mm Hg) were constricted with U46619 (30–50%) and incremental doses of acetylcholine were added to the bathing solution while recording steady state vessel diameter. Data are presented as the mean of % relaxation ± SEM. The number of experimental determinations is presented in parenthesis. *P < .05, compared to lean rats, ^+^ *P* < .05 compared to untreated obese Zucker rats.

Data in Figure 2 demonstrate that acetylcholine-mediated vascular relaxation is decreased in epineurial arterioles of the sciatic nerve from obese Zucker rats at 32 weeks of age compared to lean rats. Relaxation to maximal dosage of sodium nitroprusside (10^−4^ M) was also significantly decreased in obese Zucker rats compared to lean control rats (89.6 ± 4.4% and 58.8 ± 6.7%, resp.). Treating obese Zucker rats at 20 weeks of age with AVE7688 for 12 weeks significantly reduced the extent of vascular impairment.

**Figure 3:**
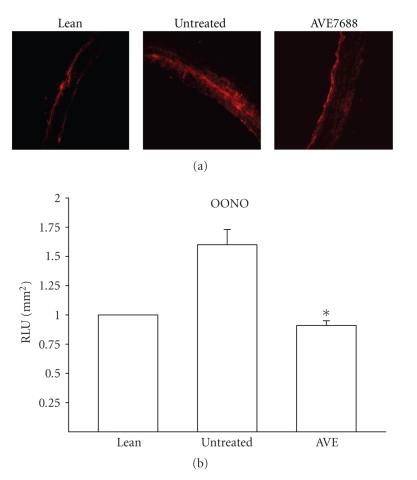
Determination of aorta superoxide and nitrotyrosine in epineurial arterioles of the sciatic nerve from lean and obese Zucker rats treated with AVE7688. Presented are representative fluorescent photomicrographs of confocal microscopic sections of aorta for nitrotyrosine staining. Lucigenin-based assay was used to determine superoxide levels in the aorta. The graphed data are presented as arbitrary light units with the value for lean rats assigned as 1. *P < .05 compared to untreated obese Zucker rats. These values were obtained from 2 different rats and 5 vessel segments were analyzed for each individual rat.

In order to obtain information on the oxidative stress status of the vasculature of untreated and treated obese Zucker rats we performed analysis of superoxide levels and nitrotyrosine staining of the aorta. The data obtained with the aorta does not necessarily reflect the oxidative stress conditions of epineurial arterioles but does serve as a marker of oxidative stress in vascular tissue and the effect of treatment. Data in Figure 3 demonstrate that superoxide levels and nitrotyrosine staining are increased in aortas of obese Zucker rats compared to lean rats at 32 weeks of age. Treating obese Zucker rats with AVE7688 significantly reduced the level of superoxide and nitrotyrosine staining in the aorta.

## 4. Discussion

We previously reported that obese Zucker rats develop vascular and neural impairment independent of hyperglycemia [9]. Furthermore, we demonstrated that impairment in vascular relaxation to acetylcholine in epineurial arterioles of the sciatic nerve preceded the slowing of nerve conduction velocity [9]. We have made similar observations in streptozotocin-induced diabetic rats and ZDF rats [35, 42, 43]. In these diabetic rat models vascular and neural dysfunctions appeared earlier than in Zucker rats, which we attribute to the influence of hyperglycemia. However, the observation that vascular and neural dysfunctions can occur in the absence of hyperglycemia is important and could have implications in patient care if these results are translatable to humans. In this regard, it has been shown that small fiber neuropathy exists in patients with metabolic syndrome and may be an early marker of sensory neuropathy [44]. The neuropathy associated with impaired glucose tolerance is milder than the neuropathy associated with diabetes but has been syndromically linked to the typical painful polyneuropathy seen in diabetic patients [45, 46].

Based on our previous results the obese Zucker rat is an appropriate animal model to study development and progression of neuropathy independent of hyperglycemia [9]. The current study demonstrate that the sensory neuropathy that occurs is characterized by a slowing of sensory nerve conduction velocity, thermal hypoalgesia and impaired tactile response when obese Zucker rats are 32 weeks of age. It is unknown whether obese Zucker rats at an earlier age experience thermal hyperalgesia as been shown in early stages of diabetic neuropathy in streptozotocin treated rats [31, 47]. Furthermore, it is unknown whether obese Zucker rats at later stages experience a loss in intraepidermal nerve fiber density, which may explain the decrease in pain perception in the hindpaw. The answer to these questions will require further study.

Given that obesity and prediabetes within the population is reaching epidemic levels there is a need to identify effective therapies for complications that occur with these conditions. Vasopeptidase inhibitors are a new class of drug that inhibits both angiotensin converting enzyme and neutral endopeptidase. Thus, these drugs have the potential to enhance vasodilation properties of peptides that are degraded by neutral endopeptidase such as the natriuretic peptides, bradykinin, calcitonin gene related peptide and adrenomedullin as well as inhibit the production of the vasoconstrictor angiotensin II [10, 18]. In several hypertension prone rat models vasopeptidase inhibitors have been shown to reduce hypertension and cardiac hypertrophy [17, 18, 48, 49]. In ZDF rats, a model for type 2 diabetes, and spontaneously hypertensive rats (SHR) vasopeptidase inhibitor treatment prevented nephropathy [22, 23, 50]. Our previous studies demonstrated that treating streptozotocin-induced diabetic rats and ZDF rats with AVE7688 improved vasodilation of epineurial arterioles of the sciatic nerve, nerve conduction velocity and blood flow in the sciatic nerve [20, 21].

In the current study we examined the efficacy of AVE7688 on vascular and neural dysfunctions in obese Zucker rats. Treatment was started at 20 weeks of age and studies performed when the rats were 32 weeks of age. At 20 weeks of age vascular relaxation to acetylcholine by epineurial arterioles is impaired but motor nerve conduction velocity is normal [9]. By 32 weeks of age both vascular and neural functions were impaired [9]. Treating obese Zucker rats with AVE7688 improved both vascular and neural dysfunctions and responsiveness to a painful stimulus.

The improvement in vascular function may be due to several mechanisms. First, there is an increase in superoxide and nitrotyrosine staining in vascular tissue of obese Zucker rats [9]. This could lead to quenching of nitric oxide one of two mechanisms responsible for acetylcholine-mediated vascular relaxation in epineurial arterioles [39]. Treating obese Zucker rats with AVE7688-reduced markers of oxidative stress in vascular tissue. Second, we previously demonstrated that acetylcholine induced vascular relaxation in epineurial arterioles is also mediated by the formation of endothelium-derived hyperpolarizing factor (EDHF) and that C-type natriuretic peptide (CNP) meets several of the criteria of EDHF in these vessels [19, 38, 50]. We have shown that epineurial arterioles express CNP and that CNP-induced vasodilation of epineurial arterioles is decreased by diabetes [20]. Treating diabetic rats with AVE7688 protected acetylcholine- and CNP-mediated vasodilation of epineurial arterioles [20]. Thus, protection of CNP from degradation could also contribute to improved acetylcholine-mediated vascular relaxation in epineurial arterioles from obese Zucker rats. However, it should be noted that the impairment of vascular relaxation to acetylcholine was not completely prevented by treating obese Zucker rats with AVE7688. This contrasts with our studies in streptozotocin-induced diabetic rats and ZDF rats [20, 21]. In those studies treatment with AVE7688 completely restored acetylcholine-mediated vascular relaxation. This could be due to other factors such as hyperlipidemia contributing to vascular impairment in obese Zucker rats. In a previous study we found that treating obese Zucker rats with Enalapril or Rosuvastatin improved acetylcholine-mediated vascular relaxation in epineurial arterioles to a similar extent as AVE7688. In that study as well as in this study treatment was not able to fully correct the hyperlipidemia [51]. A more aggressive therapy aimed at lowering lipids in combination with AVE7688 may be necessary to completely restore vascular function in epineurial arterioles.

It has been demonstrated that treating obese Zucker rats with mixanpril, a vasopeptidase inhibitor, improves insulin sensitivity in obese Zucker rats [52, 53]. In our studies treating obese Zucker rats with AVE7688 increased serum insulin levels at 32 weeks of age. Previously, we demonstrated that serum insulin levels peak at about 16 weeks of age and then gradually decline to levels observed in lean rats at about 40 weeks of age [9]. Treating obese Zucker rats with AVE7688 maintained serum insulin at levels comparable to those observed in a younger animal. Insulin resistance is an important risk factor for endothelial dysfunction [54]. It has been shown that improving insulin sensitivity improves vascular resistance in obese Zucker rats [55]. Insulin has also been shown to be a vasodilator in many vessel beds and causes vascular relaxation in epineurial arterioles of the sciatic nerve (unpublished results). Therefore, the increase in insulin levels could have contributed to the overall improved vascular function seen in obese Zucker rats treated with AVE7688 and this could improve blood flow to peripheral nerves and thereby nerve function.

Motor and sensory nerve conduction velocity as well as behavioral responsiveness to a painful stimulus was significantly improved by treatment of obese Zucker rats with AVE7688. This was similar to the effect of AVE7688 treatment of streptozotocin-induced diabetic rats and ZDF rats and Enalapril treatment of obese Zucker rats [20, 21, 51]. Comparing the overall efficacy of Enalapril and AVE7688 treatment of obese Zucker rats we find not much of a difference [51]. Both drugs appear to be effective to the same extent in improving vascular and nerve dysfunctions in this animal model. We previously concluded that AVE7688 treatment of diabetic rats was more efficacious then Enalapril [56]. The reason for this difference is unknown but may be due to the greater role hyperlipidemia may have on complications in the obese Zucker rat.

It is intriguing to speculate on the mechanism responsible for thermal hypoalgesia in 32-week-old obese Zucker rats. This is the first report of impaired pain perception in obese Zucker rats. As stated above, we do not know at this time whether intraepidermal nerve fiber density is decreased at this age in obese Zucker rats. However, it has been reported that in skin biopsies from patients with metabolic sydrome there is a decrease in mean dendrite length [44]. In streptozotocin-, cisplatin-, and paclitaxel-induced neuropathy slowing in sensory nerve conduction velocity correlated with intraepidermal nerve fiber density quantification [57]. Thus, a decrease in intraepidermal nerve fiber density in older obese Zucker rats would not be surprising and could explain the decrease in pain perception in these rats. Our finding that obese Zucker rats are less sensitive to a mechanical stimulus differs from previous studies in streptozotocin-diabetic rat models where tactile allodynia is a common finding [31, 32, 47]. The reason for this difference between these models is unknown and requires confirmation. However, such a finding could have important implications in patient care and management.

## 5. Conclusions

The important findings from this study was that 12 weeks of treatment of obese Zucker rats, an animal model of metabolic syndrome, with AVE7688, a vasopeptidase inhibitor that simultaneously inhibits angiotensin converting enzyme and neutral endopeptidase activity, improves microvascular and nerve functions as well as impaired pain perception in animals 32 weeks of age. This new class of drug that has been previously shown to improve cardiovascular and renal dysfunctions may be an effective approach for the treatment of a number of complications associated with metabolic syndrome and diabetes. 

## Figures and Tables

**Table 1:** Effect of obesity and treatment with AVE7688 on body weight, blood glucose, serum insulin, cholesterol, triglycerides, free fatty acids, and ACE activity, and thermal nociception and tactile allodynia.

Determination	Lean (17)	Obese (9)	Obese + AVE7688 (14)
Start weight (g)	190 ± 4	216 ± 7	216 ± 7
End weight (g)	522 ± 9	785 ± 33*	842 ± 21*
Blood glucose (mg/dL)	84 ± 5	96 ± 9	116 ± 15
Insulin (ng/mL)	7.7 ± 2.5	16.7 ± 1.5*	71.6 ± 19.3*^+^
Cholesterol (mg/mL)	6.0 ± 0.6	18.0 ± 3.5*	15.9 ± 4.0
Triglycerides (mg/dL)	2.53 ± 0.40	13.60 ± 2.14*	8.21 ± 1.68*
Free fatty acids (mmol/L)	0.13 ± 0.04	0.63 ± 0.23*	0.22 ± 0.03
ACE activity (U/l)	37.5 ± 3.4	211.2 ± 26.4*	38.0 ± 5.7^+^
Thermal nociception (sec)	12.8 ± 0.4	20.3 ± 0.8*	15.4 ± 0.9^+^
Tactile 50% response threshold (g)	9.8 ± 1.2	16.6 ± 1.6*	10.1 ± 1.3^+^

Data are presented as the mean ± SEM. *P < .05 compared to lean, ^+^ *P* < .05 compared to obese. Parentheses indicate the number of experimental animals.
